# Editorial: From Meaning of Working to Meaningful Lives: The Challenges of Expanding Decent Work

**DOI:** 10.3389/fpsyg.2016.01119

**Published:** 2016-07-27

**Authors:** Annamaria Di Fabio, David L. Blustein

**Affiliations:** ^1^Department of Education and Psychology (Psychology Section), University of FlorenceFlorence, Italy; ^2^Department of Counseling, Developmental, and Educational Psychology, Boston CollegeChestnut Hill, MA, USA

**Keywords:** meaning of work, meaningful lives, decent work, relationships, challenges

This Research Topic explores issues that are central to the continued relevance of organizational and vocational psychology, and equally central to the well-being of individuals and communities. The cohering theme of this publication revolves around the question of how people can establish meaningful lives and meaningful work experiences in light of the many challenges that are reducing access to decent work. Numerous interrelated trends are reshaping access to work, especially stable, dignified jobs. These trends include the continued fallout from the Great Recession, which has had a profound impact on many regions of the world (International Labour Organization, 2016). Simultaneously, the rapid growth in information technology is reducing the number and quality of jobs for people across the occupational spectrum (Brynjolfsson and McAfee, 2014; Standing, 2014).

Another essential contextual factor that is explored in this volume is the Decent Work Agenda (International Labour Organization, 2008), which represents an initiative by the International Labour Organization. In this book, we hope to enrich the Decent Work Agenda by infusing the knowledge and perspectives of psychology into contemporary discourses about work, and well-being.

During this period when work is changing so rapidly, leaving people yearning for a sense of connection and meaning, we believe that scholarship is needed to foster new developments in understanding how people construct meaning about their lives and work. This new era calls for an innovative perspective in constructing decent work and decent lives: the passage from the paradigm of motivation to the paradigm of meaning, where the sustainability of the decent life project is anchored to a meaningful construction.

Another inspiration for this project emerged from the UNESCO Chair in Lifelong guidance and counseling, recently established in Poland in 2013 under the leadership of Jean Guichard, which has focused on advancing research and policy advocacy about decent work. After the inaugural Conference in November 2013 in Wroclaw (Poland), the following Conference was in Florence in 2015 on the topic “How can career and life designing interventions contribute to a fair and sustainable development and to the implementation of decent work over the world?”

In the section that follows, we summarize the articles that are included in this volume, highlighting their connections to the main themes of this project.

The 1st article by Blustein et al. summarized existing scholarship on decent work; using the psychology of working framework (Blustein, 2006, 2013), the authors created a framework for an explicitly psychological analysis of decent work.

The 2nd article by Di Fabio and Maree proposed a transdisciplinary interpretive lens as a means of understanding the psychological meaning of decent work. This article provided a thoughtful synthesis of legal, philosophical, economic, sociological and psychological perspectives of poverty and decent work, creating important theoretical linkages.

The 3rd article by Pouyaud used the decent work lens to explicate a career counseling intervention. Pouyaud reviewed the literature on decent work and built important theoretical linkages between decent work and vocational psychology.

The 4th article by Di Fabio and Kenny presents a new theoretical model entitled Positive Self and Relational Management (PS&RM) for the Twenty-first century. Using a structural equation model design, this theoretical model explored three interrelated constructs, Positive Lifelong Life Management, Positive Lifelong Self-Management, and Positive Lifelong Relational Management into a coherent framework.

The 5th article by Ribeiro et al. examined the concept of decent work via narratives derived from 20 non-college educated adults in Brazil. Ribeiro et al. demonstrated that the concerns conveyed by the participants paralleled the attributes of decent work.

The 6th article by Allan et al. used self-determination theory in conjunction with the psychology of working framework in study that identified predictors of meaning at work. Their findings underscored the importance of internal motivation as a major predictor of meaningfulness at work.

The 7th article by Luke et al. reported a qualitative study that involved interviews with 22 retirees who returned to work after a period of retirement. Using Career Construction Theory (Savickas, 2002) and the psychology of working framework (Blustein, 2006), Luke et al. affirmed the importance of work as means of establishing meaning in life for older adults.

The 8th article by Di Fabio and Gori described the development of a new construct and measurement tool for assessing workplace relational civility (WRC). This measure provides a means of explicating an important aspect of decent work—the relational health of working people.

The 9th article by Kenny et al. examined the experiences of 18 urban, low-income graduates of a U.S. Catholic high school that was characterized by a work-place learning program. The interviews revealed that the students' experiences after high school were often marred by their exposure to external barriers.

The 10th article by Maree and Twigge used life-design theory as a framework for an analysis of career interventions provided to six adults in South Africa. Maree and Twigge identified how the life design counseling enhanced self-knowledge and also fostered growth in the relational-moral self.

The 11th article by Di Fabio and Bucci sought to explore the predictors of high school students' interests in nature and the environment. Using a quantitative design, this study found that empathy predicted connectedness to nature and affirmed that green positive counseling is optimally best understood from a relational perspective.

The 12th article by Di Fabio and Palazzeschi presented a case study that provided an illuminating perspective in the world of precarious workers. Using a life-design approach, the authors described how a 23-year old University graduate struggled to find a life of meaning while searching for stable work.

The 13th article by Ryba et al. described the development of a cultural transition model that emerged from a qualitative analysis of professional athletes who changed geographic regions in pursuing their careers.

The last article by Arnoux-Nicolas et al. presented the results of a quantitative study of 336 French workers who were surveyed about their turnover intentions, where meaning of work was inversely predictive of working conditions and turnover intentions.

## Author contributions

All authors listed, have made substantial, direct and intellectual contribution to the work, and approved it for publication.

### Conflict of interest statement

The authors declare that the research was conducted in the absence of any commercial or financial relationships that could be construed as a potential conflict of interest.
